# Organic Electroactive Molecule-Based Electrolytes for Redox Flow Batteries: Status and Challenges of Molecular Design

**DOI:** 10.3389/fchem.2020.00451

**Published:** 2020-06-19

**Authors:** Fangfang Zhong, Minghui Yang, Mei Ding, Chuankun Jia

**Affiliations:** ^1^College of Materials Science and Engineering, Changsha University of Science & Technology, Changsha, China; ^2^National Engineering Laboratory of Highway Maintenance Technology, School of Traffic & Transportation Engineering, Changsha University of Science & Technology, Changsha, China; ^3^Key Laboratory of Advanced Energy Materials Chemistry (Ministry of Education), Nankai University, Tianjin, China

**Keywords:** organic electrolyte, molecular design, redox flow battery, energy storage, electrochemistry

## Abstract

This is a critical review of the advances in the molecular design of organic electroactive molecules, which are the key components for redox flow batteries (RFBs). As a large-scale energy storage system with great potential, the redox flow battery has been attracting increasing attention in the last few decades. The redox molecules, which bridge the interconversion between chemical energy and electric energy for RFBs, have generated wide interest in many fields such as energy storage, functional materials, and synthetic chemistry. The most widely used electroactive molecules are inorganic metal ions, most of which are scarce and expensive, hindering the broad deployment of RFBs. Thus, there is an urgent motivation to exploit novel cost-effective electroactive molecules for the commercialization of RFBs. RFBs based on organic electroactive molecules such as quinones and nitroxide radical derivatives have been studied and have been a hot topic of research due to their inherent merits in the last decade. However, few comprehensive summaries regarding the molecular design of organic electroactive molecules have been published. Herein, the latest progress and challenges of organic electroactive molecules in both non-aqueous and aqueous RFBs are reviewed, and future perspectives are put forward for further developments of RFBs as well as other electrochemical energy storage systems.

## Introduction

### Energy Storage Systems

To decrease the use of fossil fuels and the emission of CO_2_, which causes global warming, the development of renewable and clean energy has become an urgent issue in many research fields, particularly in energy source (Turner, 1999; Dunn et al., 2011; Soloveichik, 2015) and eco-environmental (Yang et al., 2011) studies. Solar and wind energy are environmentally friendly and are regarded as promising alternative energy resources for the future. However, inherent intermittency and instability limit the market penetration of these renewable resources (Holdren, 2007; Dunn et al., 2011; Yang et al., 2011). Energy storage technology, which is an effective solution for the intermittency, is crucial for the expansion of renewable energy (Armand and Tarascon, 2008; Dunn et al., 2011; Yang et al., 2011; Leadbetter and Swan, 2012; Ding et al., 2013; Zhang et al., 2019). So far, the most common approach for grid-scale energy storage is a pumped hydro-electric system, which exhibits noticeable geographic constraints. Consequently, exploiting new energy storage technology with high performance and flexible design has been one of the top topics of research in the past few decades. Electrochemical energy storage systems, also known as rechargeable batteries (secondary battery), which use redox-active molecules to fulfill the energy conversion, are well-developed (Ding et al., 2018; Luo et al., 2019) and offer highly efficient energy conversion and smart design feasibility. Examples are lead-acid batteries, lithium-ion batteries, supercapacitors, and redox flow batteries (RFBs) (Schon et al., 2016; Ding et al., 2018).

Due to its low capital cost and mature technological support, the lead-acid battery was employed for massive energy storage and dominated the electrochemical energy storage market in the twentieth century (Yang et al., 2011). However, the limited cycling lifetime, the high maintenance cost, and the severe contamination caused by lead contributed to it being replaced by other electrochemical approaches (Ma et al., 2015; Liu et al., 2018; Yang et al., 2018a). Nowadays, the most dominant electrochemical energy storage pathway is the lithium-ion battery in which lithium (Li^+^) ions shuttle between the positive and negative electrodes (Xu et al., 2015; Chen et al., 2017). During the cycling process, Li^+^ ions repeatedly take part in intercalation/de-intercalation at the two electrodes in a round-trip fashion. During the charging process, Li^+^ ions de-intercalate from the positive electrode, migrate across the separator, and then intercalate into the negative electrode, accompanied by oxidation and reduction reactions at the positive and negative electrode, respectively. During discharging, the reverse processes occur (Wei et al., 2018). To tackle the challenges that the lithium-ion battery faces, researchers have done extensive, in-depth investigations into improvements in the electrodes, electrolytes, and separator materials. Prior work has pointed out the electrolyte can work in liquid, gel, and solid states (Li et al., 2015b, 2016b). Liquid electrolytes, which incorporate organic alkyl carbonate solvents, for instance, ethylene, dimethyl, diethyl, and ethyl methyl carbonate, with dissolved lithium salts like LiBF_4_, LiClO_4_, LiPF_6_, LiBC_4_O_8_, and Li[PF_3_(C_2_F_5_)_3_], are most widely used due to their good fluidity and ionic conductivity (Chen et al., 2013; Li et al., 2015c). Due to the advances such as high energy density and long cycling lifetime that have been achieved, lithium-ion batteries have exerted enormous, and dramatic effects on society (Li et al., 2015a,d; Chen et al., 2019; Yang et al., 2019). However, there are some tough issues, such as low abundance of materials, short discharge time, and the flammability of the solvents used in the battery, to be dealt with for industrial and residential implementation of lithium-ion batteries for large-scale energy storage (Yang et al., 2011; Li et al., 2016a). Inspired by the fact that the inherent electrochemical properties of sodium are close to those of lithium and its abundant distribution on the earth, sodium-ion batteries, as well as potassium-ion batteries, are attracting increasing amounts of interest in the last few decades, though these are still in their infancy in terms of application in large-scale energy storage (Chen et al., 2018; Rajagopalan et al., 2020a,b).

Supercapacitors, which have fast charge/discharge response, high power density, and excellent cycling performance, are also regarded as a promising energy storage avenue (Qin et al., 2019; Wu et al., 2019a). Based on energy storage mechanism, supercapacitors are grouped into electrical double-layer capacitors (EDLCs) and pseudocapacitors (faradaic capacitors). EDLCs, comprised of high specific surface area materials like activated carbon, carbon nanotubes, and graphene derivatives, adsorb ions at the electrode/electrolyte interface to achieve the energy storage. Pseudocapacitors, meanwhile, adopt transition metal oxides (such as RuO_2_, MnO_2_, NiO, and Co_3_O_4_) with high theoretical specific capacity and excellent redox reversibility as electrodes to realize energy storage via redox reactions at the electrodes (Wu et al., 2018b; Zeng et al., 2019). Low energy density, poor cyclability, and high cost due to the use of precious metals limit the application of supercapacitors for storing massive amounts of energy (Wu et al., 2018a, 2019b).

Given the deficiencies of the above-mentioned technologies, to broaden the market penetration of renewable resources for electricity generation, any novel electrochemical energy storage system that operates at grid-scale needs to address both technical and economic concerns. The RFB, invented in 1974 and exhibiting merits like design flexibility, safety, and scalability, is considered a very promising pathway toward practical large-scale energy storage (Leung et al., 2017). The most striking advantage of RFBs is the independent engineering of energy (number of moles of redox materials and cell voltage) and power (electrode area) owing to the unique cell configuration (Kamat et al., 2017; Park et al., 2017). It is noted that the chemical stability, electrochemical reversibility, and reduction potentials of redox materials contribute to the cell performance of RFBs, and the cost of redox materials is associated with the system capital cost. Undoubtedly, redox materials ranging from inorganic metal ions, halogens, and polysulfides to organic electroactive molecules play a crucial role in the improvement of RFBs. In this review, the research status and advances of organic electroactive molecules in both non-aqueous and aqueous RFBs are presented, as are the challenges and future perspectives in this promising field.

### Redox Flow Batteries

**Figure 1:**
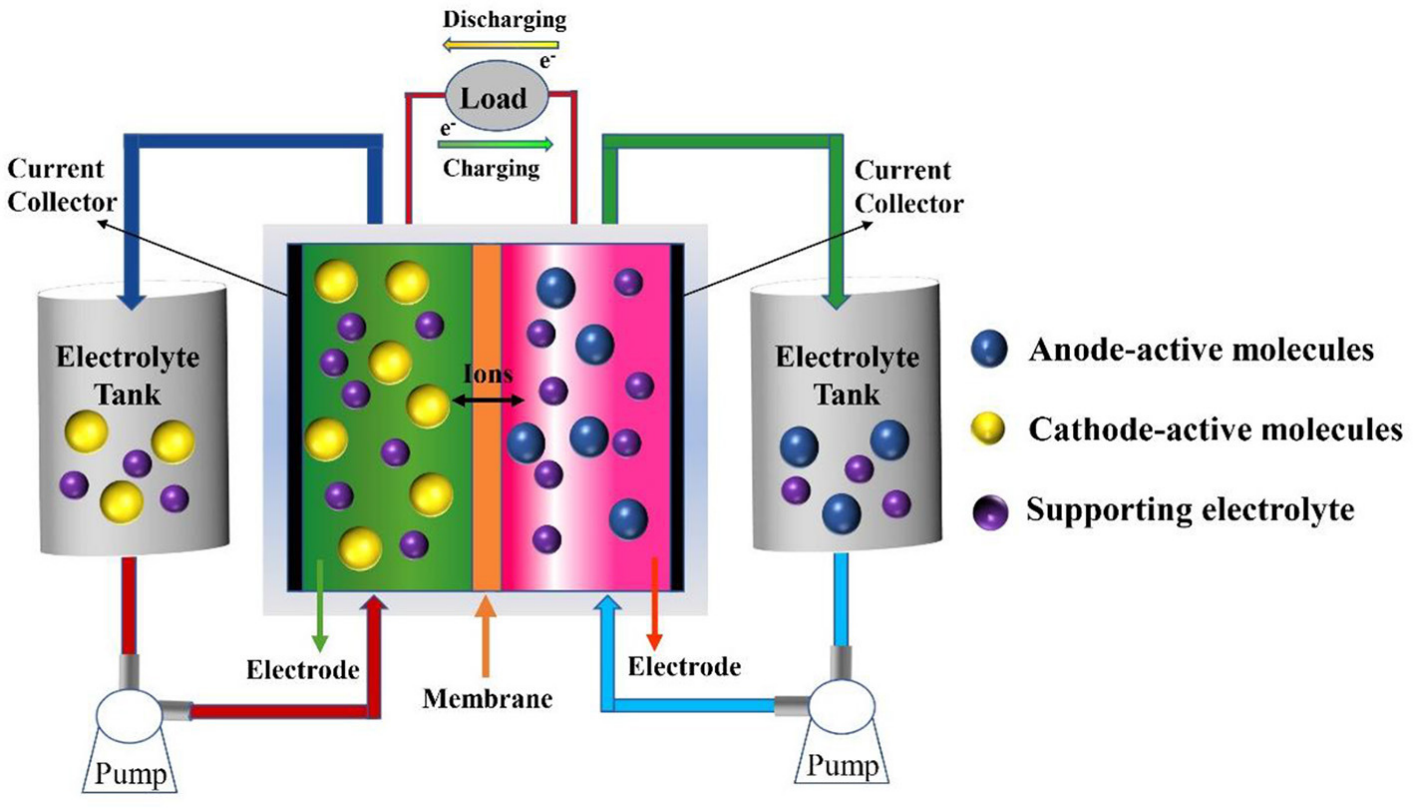
Schematic illustration of a typical redox flow battery.

The main components of RFBs include two electrolyte tanks (anodic and cathodic reservoirs), anodic-active and cathodic-active materials (anolyte and catholyte), an ion-exchange membrane, and the battery framework (Figure 1). Energy interconversion between chemical energy and electric energy is accomplished via the redox reaction of electroactive materials dissolved in supporting electrolyte, which circulates between the tanks and corresponding compartments of the electrochemical cell, driven by external pumps at the electrodes. Selective ions migrate across the ion-exchange membrane to complete the current circulation. According to the dissolving condition of electrolytes, RFBs can be divided into aqueous RFBs and non-aqueous (organic) RFBs.

The state-of-the-art RFB systems employ all-vanadium ions as electroactive materials, and the largest (200 MW/800 MWh) is under construction by Rongke Co. Ltd. Low energy density, the need for strong sulfuric acid as the supporting electrolyte, a spatially inhomogeneous distribution, and the volatile price of vanadium constrain the widespread deployment of all-vanadium RFBs. As alternative approaches, RFBs with electroactive materials based on other inorganic redox ions, such as Fe^0/2+/3+^, Cr^2+/3+^, Zn^0/2+^, Br_2_/Br^−^, I_2_/I${}_{3}^{-}$, and S${}_{\text{x}}^{2-}$ (Park et al., 2017; Ding et al., 2018; Luo et al., 2019) have been proposed and investigated. Along with advances in electrodes and the ion-exchange membrane, improvements in RFB cell performance have also been achieved to some extent (Ye et al., 2018, 2019a,b, 2020; Chang et al., 2019; Lou et al., 2019; Xia et al., 2019). However, these systems are still facing technological challenges, such as the high viscosity of a strong sulfuric acid supporting electrolyte, dendritic deposition of zinc, the strong corrosivity of halogen, high cost, and so on, hindering practical application. Organic redox molecules, which have been known of for a long time, have attracted increasing interest in the field of synthetic chemistry, catalysts, functional materials, and industrial applications. Given their inherent redox nature, the low cost of raw starting materials, and the flexibility of their chemical and physical properties (solubility, stability, redox potential, et al.) when tailored by substituents, organic redox molecules, such as anthraquinone derivatives, nitroxide radicals, and polymers, have been adopted for energy storage materials to construct high cell performance and cost-effective RFB systems in the past decade. Depending on the supporting electrolytes, RFBs with organic electroactive materials can be simply classified as aqueous and non-aqueous systems, respectively.

### Design Principles of Organic Electroactive Molecules

#### Physicochemical and Electrochemical Properties

It is worth noting that the energy density of RFBs is dependent on the number of electrons involved in the electrode reaction, the concentration of electroactive molecules in the electrolytes, and the potential difference between the cathodic and anodic active molecules.

The solubility limit, corresponding to the highest concentration of organic electroactive molecules possible, which varies with solvent, needs to be as high as possible. As pointed out in prior work, the physical properties of the solvent, such as the pH value, viscosity, polarity, and dielectric constant, have huge impacts on the solubility limit (Ding et al., 2018; Luo et al., 2019). Moreover, the supporting electrolyte in the same solvent changes the solubility of the redox molecule as well as the internal resistance of a cell. The organic electroactive molecule, solvent, and supporting electrolyte should be taken into account together to develop an RFB with high energy efficiency and coulombic efficiency. Given a large variety of solvents consisting of aqueous and non-aqueous solvents, a higher solubility limit for organic electroactive molecules can be achieved than for inorganic redox materials. In other words, under the same solvent and supporting electrolyte conditions, taking advantage of flexible modification by substituents of organic molecules, the concentration can be increased as envisioned. Based on the rule that likes dissolve each other, molecules with higher relative permittivity in solvents can increase the solubility as well as the stability. Thus, in aqueous RFBs, water-soluble ionic or polar substituents such as the quaternary ammonium, sulfonic, carboxyl, and hydroxy groups can be employed to increase the concentration of organic electroactive molecules. As for the organic electroactive molecules in non-aqueous RFBs, fat-soluble substituents such as alkyl, carbonyl, and ester groups can be helpful to increase the solubility limitation. The desirable values of solubility of redox species in aqueous solvents are around 1–2 M, while in non-aqueous electrolytes 4–5 M is required to satisfy the demand for cost-effective energy storage (Darling et al., 2014).

The formal cell voltage of an RFB is obtained by the difference in reduction potential between the cathodic and anodic active molecules. As reported in previous work, the reduction potential of organic electroactive molecules can be shifted more to the positive or negative side by the electrostatic properties and positions of functional groups on the backbone (Burgess et al., 2018). The cell voltage of aqueous RFBs is constrained by the electrolysis of water, with a potential window limit of 1.24 V. Freedom from problems caused by water electrolysis enables non-aqueous RFBs to reach high cell voltages, which compensates for the higher concentration requirement in view of economic viability. Commonly, within the potential window of the solvent, a higher cell voltage can be designed and obtained with a non-aqueous organic RFB than with an aqueous organic RFB. Moreover, the lifetime duration is another important indicator parameter for the performance of an RFB. The chemical and electrochemical stability of organic redox species both play a crucial role in the cycling lifetime. The proposed decomposition mechanisms of organic electroactive materials used in RFBs have been summarized in the latest review (Kwabi et al., 2020), namely nucleophilic addition and hydrolysis, disproportionation, dimerization, and tautomerization. Hence, according to the inherent nature of organic molecules, by rational substituent design to prevent the redox core from degradation, more stable electroactive materials and longer cycling time can be realized.

#### Chemical Cost

One prerequisite for the commercialization of RFBs is low cost. Note that the grid-scale application of the well-known all-vanadium RFB is hindered by the low earth-abundance and volatile price of vanadium. Because they utilize organic molecules consisting of high earth-abundance elements such as carbon, hydrogen, oxygen, nitrogen, the capital cost of most RFBs with organic electroactive materials can drastically drop to the expected value for practical applications. Despite this, some special organic electroactive materials such as radicals still cost much more than common inorganic electroactive species. By screening the reported RFBs with organic redox couples, it has been found that synthesis of organic materials must be performed under an inert atmosphere or even in an argon or nitrogen-purged glovebox to avoid side reactions with oxygen from the ambient atmosphere. The use of such harsh synthesis conditions, along with the requirement of catalysts comprised of noble metals, is not a cost-effective strategy. According to the target of $ 150/kWh for industrial application of RFB systems by the year of 2023 set up by the Department of Energy of the United States, cost estimates for electroactive materials of under a standard of $5/kg are desired (Darling et al., 2014). The development of robust organic electroactive molecules that can be derived from cheap, commercially available raw materials with easy-to-handle synthetic steps must be targeted to enable low-cost RFB systems.

#### Non-Aqueous Organic RFBs

Non-aqueous organic RFBs employ organic electroactive molecules and supporting electrolytes in organic solvents, such as butyrolactone, acetonitrile (ACN), dimethylacetamide (DMA), and propylene carbonate (PC). The first non-aqueous organic RFB was reported by Matsuda and co-workers in 1988, in which a ruthenium complex [Ru(bpy)_3_](BF_4_)_2_ (bpy = 2,2′-bipyridine) served as the energy storage material in acetonitrile solution with an open-circuit voltage of 2.60 V (Matsuda et al., 1988). Since conventional aqueous RFBs have a limit of the cell voltage window of 1.24 V, more and more research interest is shining a light on non-aqueous systems to pursue higher voltage output from RFBs. Moreover, non-aqueous organic RFBs exhibit other advantages, such as higher energy density, wider operating temperature range, faster reaction kinetics, and more combinations of organic redox couples. Organic electroactive molecules employed in non-aqueous organic RFBs can be classified into four groups, namely: coordination compounds and organometallic complexes, quinones, radicals, and polymers (Ding et al., 2018; Luo et al., 2019).

#### Coordination Compounds and Organometallic Complexes

**Figure 2:**
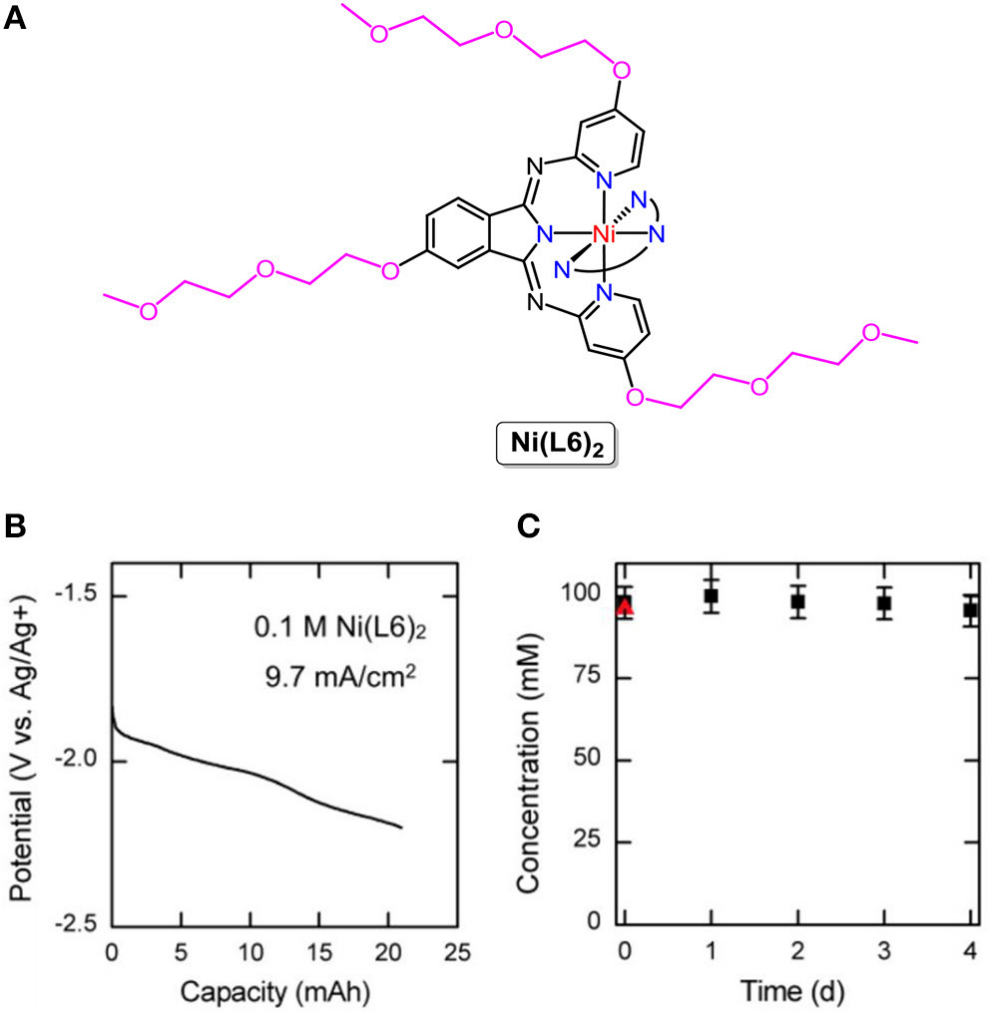
**(A)** Molecular structure of **Ni(L6)**_**2**_. **(B)** Charging potential curve for a 2e reduction of a 100 mM solution of **Ni(L6)**_**2**_. **(C)** Concentration of the charged **Ni(L6)**_**2**_ as a function of time. The red triangle represents the concentration of the neutral solution before cycling. Reproduced with permission from Sevov et al. (2016). Copyright 2016, American Chemical Society.

Coordination complexes, consisting of metal ions ligated with ligands, are the earliest-studied organic electroactive molecules in non-aqueous RFBs. [Ru(bpy)_3_](BF_4_)_2_, Ru(acac)_3_ (acac = acetylacetonate) (Chakrabarti et al., 2007), Cr(acac)_3_ (Liu et al., 2010), [Ni(bpy)_3_]^0/2+^, [Fe(bpy)_3_]^2+/3+^ (Kim et al., 2011), Mn(acac)_3_ (Sleightholme et al., 2011), VO(acac)_2_ (Herr et al., 2013), [V(mnt)_3_]^2−^ (mnt = (NC)_2_C_2_S${}_{2}^{2-}$) (Cappillino et al., 2014), [Fe(phen)_3_]^2+/3+^, [Co(phen)_3_]^2+/3+^ (phen = phenanthroline) (Xing et al., 2015), and V(acac)_3_ (Bamgbopa and Almheiri, 2017) were successively utilized to store energy in non-aqueous RFBs. Although the resulting RFBs exhibit high open-circuit voltages (1.2–3.4 V), the energy density is severely limited by the solubility of coordination complexes in organic solvents, which are mostly <1 M. By rational substituent design, the Sanford group designed and synthesized a series of coordination complexes combining earth-abundant metals with tridentate bipyridyliminoisoindoline (BPI) ligands, revealing improved physicochemical properties and cell performance. The solubility of the nickel complex abbreviated as **Ni(L6)**_**2**_ (molecular structure is shown in Figure 2A) was more than 0.7 M in acetonitrile (ACN), higher than that of other nickel complexes under the same conditions. Furthermore, the charged **Ni(L6)**_**2**_ remained stable and soluble in ACN with 0.5 M TBABF_4_ as the supporting electrolyte for days at a high concentration (0.1 M, Figure 2C). The nickel-BPI complexes could undergo over 200 charge-discharge cycles with a 5% capacity fade. It is worth noting that more than one electron was involved in the redox reactions of **Ni(L6)**_**2**_ at low redox potentials (Figure 2B), suggesting an effective way to lower the molecular weight per mole of electrons transferred and higher energy density (Sevov et al., 2016).

Ferrocene, recognized as the progenitor of organometallic chemistry, has given rise to an enormous number of ferrocene-containing ligands, molecules, and materials since its discovery in 1951 (Kealy and Pauson, 1951; Butler and Thomas, 2007; Štěpnička, 2008; Dai and Hou, 2010; Phillips, 2011; Ding et al., 2019). Synthetic flexibility, well-defined redox properties, and fast reaction kinetics make them very promising for RFBs. However, the solubility of pristine ferrocene in propylene carbonate (PC, a commonly used solvent in non-aqueous RFBs) is only 0.2 M. To address this issue, Wang and co-workers attached the Cp ring (the cyclopentene moiety of ferrocene) to different ionic charged tetraalkylammonium groups to yield a series of ferrocene derivatives. By screening the solubilities of the resulting derivatives, it was concluded that tetraalkylammonium groups could improve the solubility compared to ferrocene without any substituent. However, to some extent, the stability of ferrocene derivatives was sacrificed. For example, when PF${}_{6}^{-}$ and BF${}_{4}^{-}$ anions serve as counterions, the corresponding derivatives with enhanced solubility show poor chemical stability, which hinders further application in RFB systems (Cosimbescu et al., 2015; Wei et al., 2015). Other effective strategies like using electron withdrawing groups such as an acetyl substituent to improve the solubility of ferrocene by breaking the symmetrical electron cloud distribution of ferrocene (Kim et al., 2016) may simultaneously maintain good stability.

Although advances have been made, non-aqueous RFBs with coordination compounds and organometallic complexes still face technical and economic challenges before practical application. Concern about the utilization of precious metal catalysts in the materials synthesis process, increasing the chemical costs of RFBs, motivates efforts on investigating RFBs with metal-free organic electroactive materials.

### Quinones

Quinones are aromatic molecules with fully conjugated cyclic diketone structures and are considered the most promising electroactive molecules of the natural organic materials (Scott et al., 1998; Park et al., 2015; Jing et al., 2017). The electronegative oxygen atom draws electron density away from the carbon atom through the double bond in the carbonyl group, increasing the polarity and reactivity of the carbonyl compound. The carbonyl functional group, which acts as a redox center, and the aromatic structure, which affects the reduction potential position and stability, in combination with fast reaction kinetics, an easily modified backbone, and cost-effective synthesis processes (Häupler et al., 2015) make quinones promising candidates for RFBs (Quan et al., 2007; Huskinson et al., 2014; Er et al., 2015; Lin et al., 2015).

**Figure 3:**
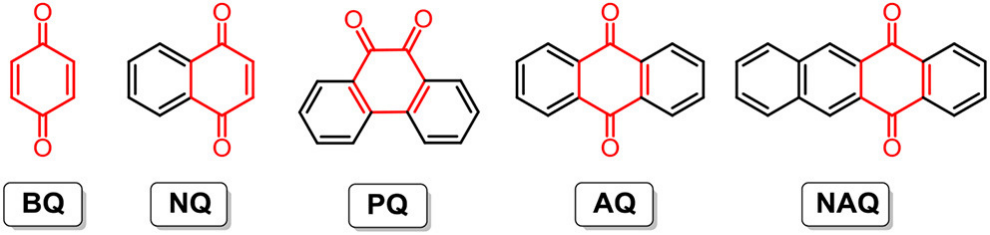
Molecular structures of quinone derivatives in non-aqueous RFBs.

It is thought that the physicochemical and electrochemical properties of quinones and the cell performance of RFBs are strongly dependent on the molecular aromaticity and electronic structures. To reveal the structure-performance correlation, the Yu group studied five quinones with regular structural variations, namely 1,4-benzoquinone (**BQ**), 1,4-naphthoquinone (**NQ**), 9,10-phenanthrenequinone (**PQ**), anthraquinone (**AQ**), and 5,12-naphthacenequinone (**NAQ**) (molecular structures are shown in Figure 3). Their experimental and computational results indicated that with the increase in aromaticity (densities of arenes) from **BQ** to **NAQ**, both the solubility and redox potential of the quinone derivatives decreased. Cell performance tests of non-aqueous RFBs with the above-mentioned quinone and derivatives show that the **NQ**-based battery reached a relatively high energy density of 60 Wh L^−1^, with nearly 100% capacity retention after 100 cycles, which can be further improved by enhancing carbonyl utilization with structural modification (Ding et al., 2016).

### Radicals

**Figure 4:**
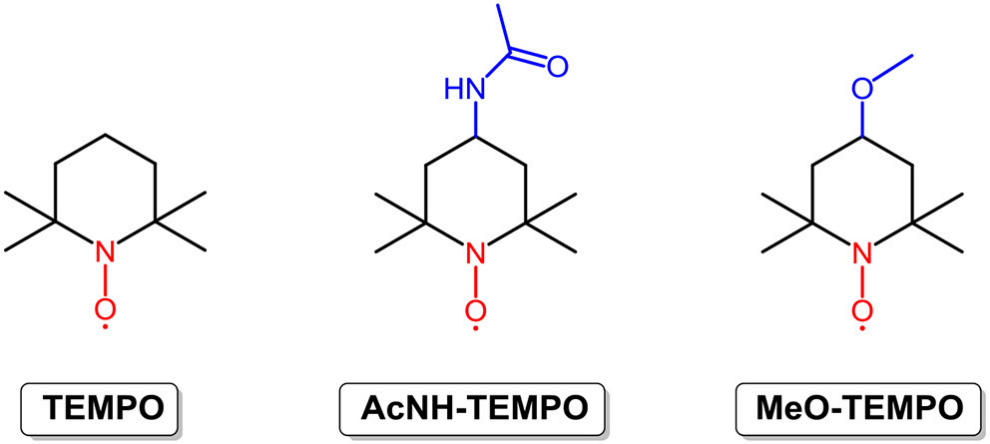
Molecular structures of **TEMPO** derivatives.

Radicals containing one or more unpaired or open-shell electrons show redox activity, enabling energy storage and conversion. However, poor stability ascribed to the formation of dimers and polymers and reactions with other molecules (Nishide et al., 2004) have become a barrier to the use of radicals in RFBs. By employing steric hindrance, resonance effects, and a stabilizer, the stability of radicals is enhanced, and the application territory is broadened. At present, radicals used for RFBs can be grouped into the following three types according to molecular construction: nitroxide radicals, alkoxybenzene-based radicals, and heterocyclic-based radicals. Among them, nitroxide radicals, as exemplified by 2,2,6,6-tetramethyl-1-piperidinyloxy (**TEMPO**; the structure is shown in Figure 4), have been widely investigated. TEMPO is a commercially available organic radical that is stabilized by the steric hindrance of four methyl groups, as well as the resonance effect between the unpaired electron and the N–O bond (hyperconjugation). With good redox reversibility and outstanding solubility in organic solvent (5.2 M in EC/PC/EMC mixture solvents), TEMPO and its derivatives have attracted increasing attention for application in RFBs (Li et al., 2011; Wei et al., 2014; Milshtein et al., 2016).

In 2011, Li et al. reported a novel all-organic redox flow battery with **TEMPO** as the positive electroactive material and 1.00 M NaClO_4_/acetonitrile as the supporting electrolyte. Stable charge-discharge curves and high coulombic efficiency (90%) were achieved in the **TEMPO**-based non-aqueous RFB (Li et al., 2011). In 2014, the Wang group prepared a high concentration solution of **TEMPO** in mixed organic solvents by the addition of excess LiPF_6_ as the supporting charge carriers. The redox potential of this **TEMPO**-based electrolyte reached 3.5 V vs. Li/Li^+^, and an RFB constructed by pairing up the **TEMPO** redox couple with the lithium redox pair output an energy density of 126 Wh L^−1^, which is approximately five times that of the all-vanadium RFBs and much higher than the majority of non-aqueous RFBs (Wei et al., 2014). It is well known that the electrochemical properties of organic molecules can be easily modified by substituents. Milshtein et al. synthesized and studied 4-acetamido-2,2,6,6-tetramethylpiperidine-1-oxyl (**AcNH-TEMPO**; the chemical structure is shown in Figure 4) to reveal the effects of an electron withdrawing functional group on the radical. The results show that **AcNH-TEMPO** exhibits a more positive reversible one-electron redox reaction at 3.63 V vs. Li/Li^+^. However, the low solubility (0.5 M in 1 M LiBF_4_/PC) of **AcNH-TEMPO** constrains the cell performance of RFBs (Milshtein et al., 2016). Inspired by this challenge, Takechi et al. employed “supercooled liquid” to improve the solubility of TEMPO derivatives, as exemplified by 4-methoxy-2,2,6,6-tetramethylpiperidine-1-oxyl (**MeO-TEMPO**; the chemical structure is shown in Figure 4). **MeO-TEMPO** was mixed with LiTFSI (lithium bis-trifluoromethanesulfonimide) at a molar ratio of 1:1, leading to the formation of a new ionic couple and “supercooled liquid.” It is worth noting that by replacing the acetamido group with a methoxy group, the resulting **TEMPO** derivative shows a comparable redox potential (3.6 V vs. Li/Li^+^) with **AcNH-TEMPO**, whereas the solubility could reach a much higher value of 2.5 M in LiTFSI/ACN solution (Takechi et al., 2015). Another limit of RFBs with radical redox materials is their high capacity loss rate, which can be conquered due to the merits of the smart design nature of organic molecules. However, the high price of TEMPO and derivatives is an obstacle to the wide deployment of **TEMPO**-based RFBs.

### Polymers

**Figure 5:**
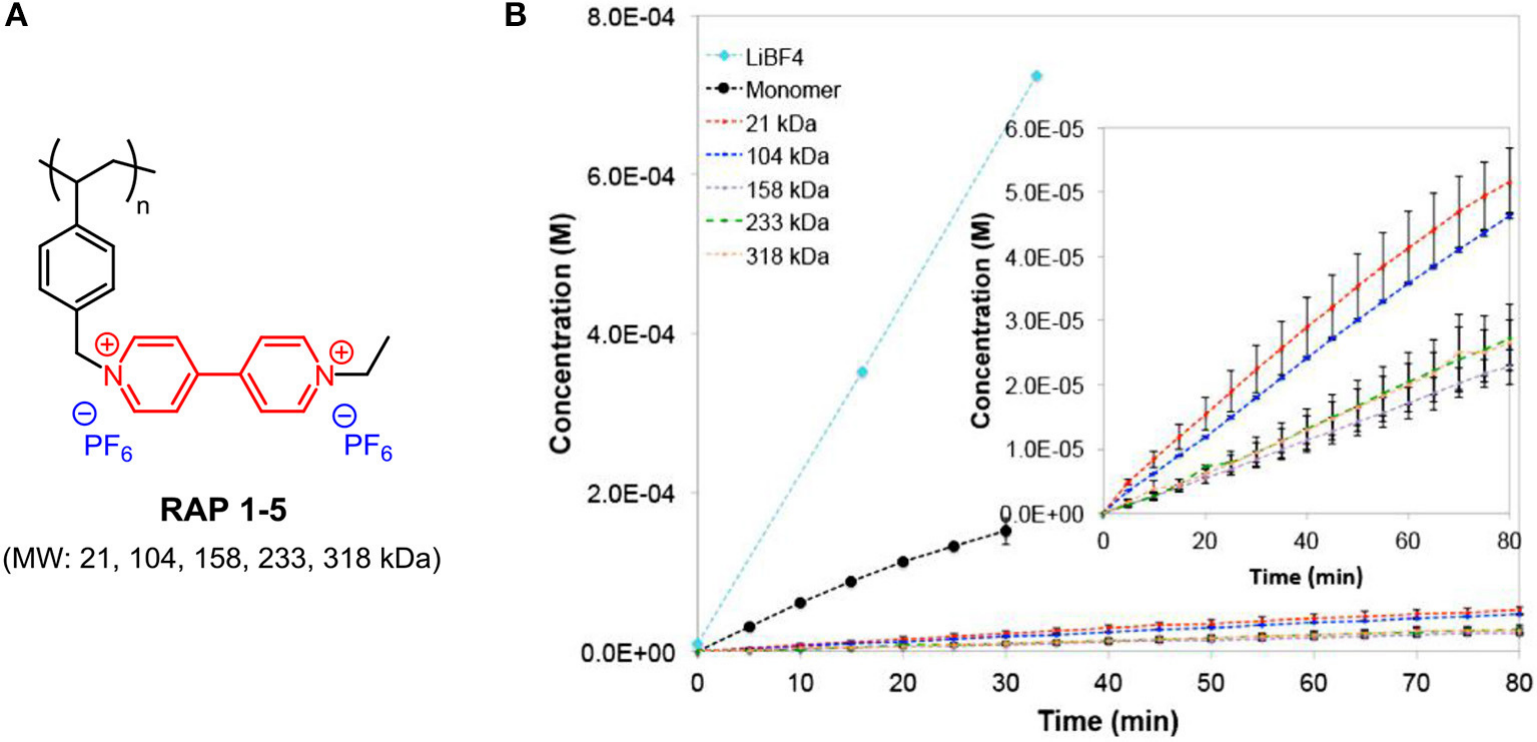
**(A)** Molecular structure of **RAPs 1-5**. **(B)** Time-dependent transport of LiBF_4_, monomer and **RAPs 1–5** across Celgard 2325 at 0.01 M. Inset shows the **RAPs 1–5** region of the plot. Reproduced with permission from Nagarjuna et al. (2014). Copyright 2014, American Chemical Society.

One common critical issue in improving the cell performance of RFBs with electrochemical small molecules is the permeability of redox species across the ion-exchange membrane, known as crossover contamination, which leads to Coulombic efficiency fade (Lai et al., 2020). It was believed that larger-sized macromolecules would have difficulty migrating through the membrane, and so electroactive polymers were employed as redox centers in RFBs (Nagarjuna et al., 2014; Burgess et al., 2016, 2018; Iyer et al., 2017). The Rodríguez-Lopez group conducted a study on size-based selective transport across commercial off-the-shelf separators for non-aqueous RFBs by preparing a series of viologen-based redox-active polymers (**RAPs 1-5**; the chemical structures are shown in Figure 5A) with different molecular weights. The severe crossover behavior of a monomer with a small molecular size through the separator could be largely suppressed by the formation of polymers (Figure 5B). It is also noted that polymer **RAPs 1-5** exhibit similar permeabilities as well as solubility, viscosity, and electrochemical properties, with negligible influence from molecular weight. For **RAPs 1-5**, good solubility (above 2.0 M in ACN and PC), electrochemical and chemical reversibility, and limited crossover contamination across porous separators make them promising energy storage materials for non-aqueous RFBs (Nagarjuna et al., 2014).

To evaluate the cell performance, the Rodríguez-Lopez group assembled RFBs employing viologen monomer or polymer and ferrocene as redox couples with inexpensive nano-porous separators and 0.1 M LiBF_4_ supporting electrolyte in acetonitrile. The results show that an RFB with polymer-based electroactive materials could achieve a high coulombic efficiency of 98% and a capacity efficiency of 80% (50 cycles). The high viscosity of polymer-containing solution slows down the mass transport of redox species and decreases the ionic conductivity of electrolyte, hindering further advances in the cell performance of RFBs. Seeking more redox polymers with improved physicochemical and electrochemical properties as well as high-performance for RFBs is an urgent tack in view of molecule design (Burgess et al., 2016; Montoto et al., 2017, 2018).

Even though much research work has been put forward, few non-aqueous RFBs have been commercialized due to their higher chemical cost, poorer cycle stability in high concentrations, and smaller ionic conductivity compared with aqueous RFBs. For non-aqueous RFBs, the ionic conductivity can be efficiently improved by increasing the concentration of organic electrolytes, which endows the non-aqueous RFBs with a higher energy density comparable to that of aqueous RFBs. However, the high concentration leads to complex side reactions in the redox cell, which lead to capacity decay after several to dozens of cycles. Bipolar molecules, which are realized by adding specific functional groups according to the environment of the redox cell and that have high solubility and enhanced chemical stability, can be employed to solve this problem. Despite their shortcomings in terms of processing, the significantly wider potential windows, extensive options for both anode and cathode-active molecules, abundance of organic solvents, high operating temperature, and faster reaction kinetics all indicate the great potential of non-aqueous RFBs for electrochemical storage in the future.

## Aqueous RFBs

Organic solvents used in non-aqueous systems are known to have disadvantages such as volatility, toxicity, corrosiveness, and flammability. When targeting residential and industrial implementation, environmentally benign aqueous RFBs are more promising. Aqueous RFBs, featuring redox species dissolved in water with supporting electrolyte, exhibit many advantages, such as high ionic conductivity, safety, cost-effectiveness, and well-developed technology to compensate for the narrow voltage window, which is limited by the electrolysis of water. Aqueous RFBs have been a hotspot in research on electrochemical energy storage in past decades. According to the pH value of electrolytes, aqueous RFBs can be grouped into three classes, namely acidic, alkaline, and neutral aqueous RFBs.

### Organic Electroactive Molecules for Acidic Aqueous RFBs

The proton, which has the smallest size (1.6 × 10^−15^ m) among cations, shows an amazingly high limiting molar conductivity of 349.8 × 10^4^ Ω^−1^ m^2^ mol^−1^ at 298 K and promises acidic aqueous RFBs with rapid charge/discharge rates and high energy efficiencies and power densities. Since the first acidic RFBs with organic electroactive molecules were reported by Huskinson et al. (2014), this approach has been attracting increasing attention (Xu et al., 2010; Lin et al., 2015; Carney et al., 2017; Li et al., 2017; Goulet and Aziz, 2018; Luo et al., 2019).

**Figure 6:**
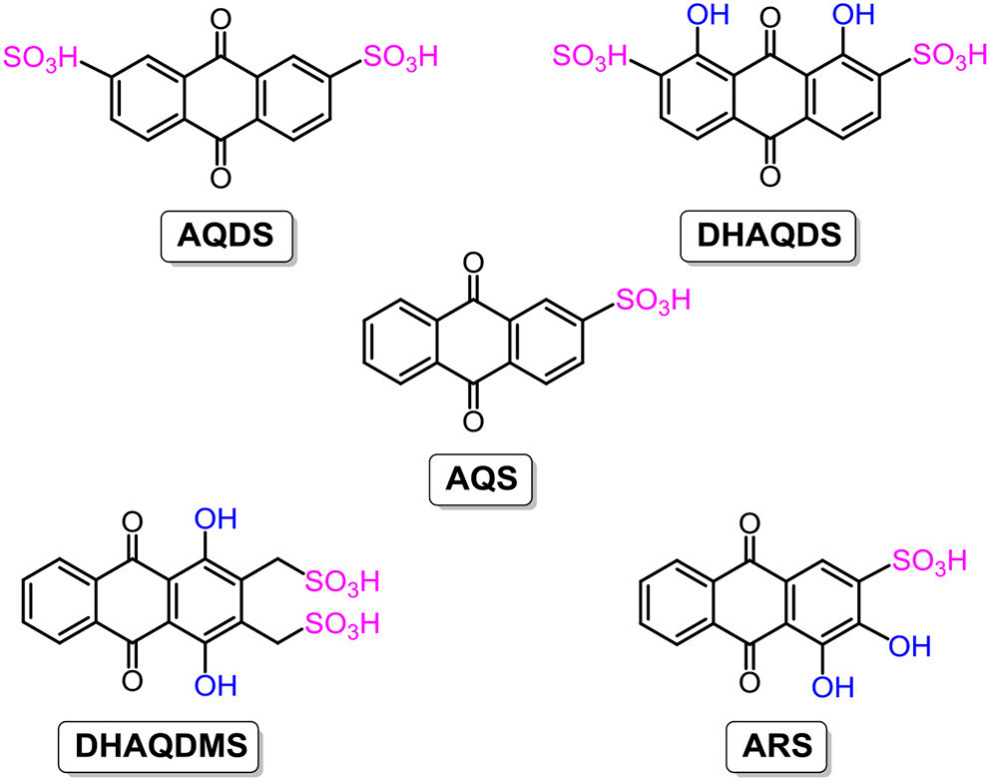
Molecular structures of anthraquinone derivatives for acidic aqueous RFBs.

Given merits like good redox reversibility, fast electron-transfer kinetics, longtime stability, and cost-effective raw materials, quinones and their derivatives are widely employed in both non-aqueous RFBs, as stated above, and aqueous RFBs. In 2014, the Aziz group constructed a new aqueous RFB with 9,10-anthraquinone-2,7-disulphonic acid (**AQDS**; the chemical structure is shown in Figure 6) as the anolyte. Thanks to the hydrophilic effect of sulfonic groups, **AQDS** shows an aqueous solubility of up to 1 M in sulphuric acid, and the resultant RFB exhibits above 99% storage capacity retention per cycle and a high energy density of 50 W h L^−1^ (Huskinson et al., 2014; Lin et al., 2016). However, in this RFB system, both the concentrated sulphuric acid serving as the supporting electrolyte and the Br^−^/Br_2_ employed as a negative electrolyte are strong corrosives, engendering extra hardware cost and maintenance fees. Besides, the low open-circuit voltage of only 0.7 V of the **AQDS**-based RFB blocks further improvements in cell performance.

Taking advantage of sulfonic groups for enhancement of the solubility of AQDS through hydrophilic effects, more substituents were introduced to study the variations in electrochemical properties. 1,8-dihydroxy-9,10-anthraquinone-2,7-disulphonic acid (**DHAQDS**; the chemical structure is shown in Figure 6) was synthesized using hydroxyl substituents. The open-circuit voltage of the **DHAQDS**-based RFB was shifted to a higher value of 1.2 V, attributed to the electron-donating effect of hydroxyl groups. Moreover, a series of anthraquinone derivatives (the chemical structures are shown in Figure 6) such as 9,10-anthraquinone-2-sulfonic acid (**AQS**), 1,4-dihydroxy-9,10-anthraquinone-2,3-dimethylsulfonic acid (**DHAQDMS**), and Alizarin Red S (**ARS**) were synthesized and characterized electrochemically to reveal the influences of the amounts and positions of substituents. As mentioned above, smart design and modifications of organic electroactive molecules is an effective approach to obtain high-performance RFBs aimed at large-scale energy storage (Gerhardt et al., 2017).

### Organic Electroactive Molecules for Alkaline Aqueous RFBs

**Figure 7:**
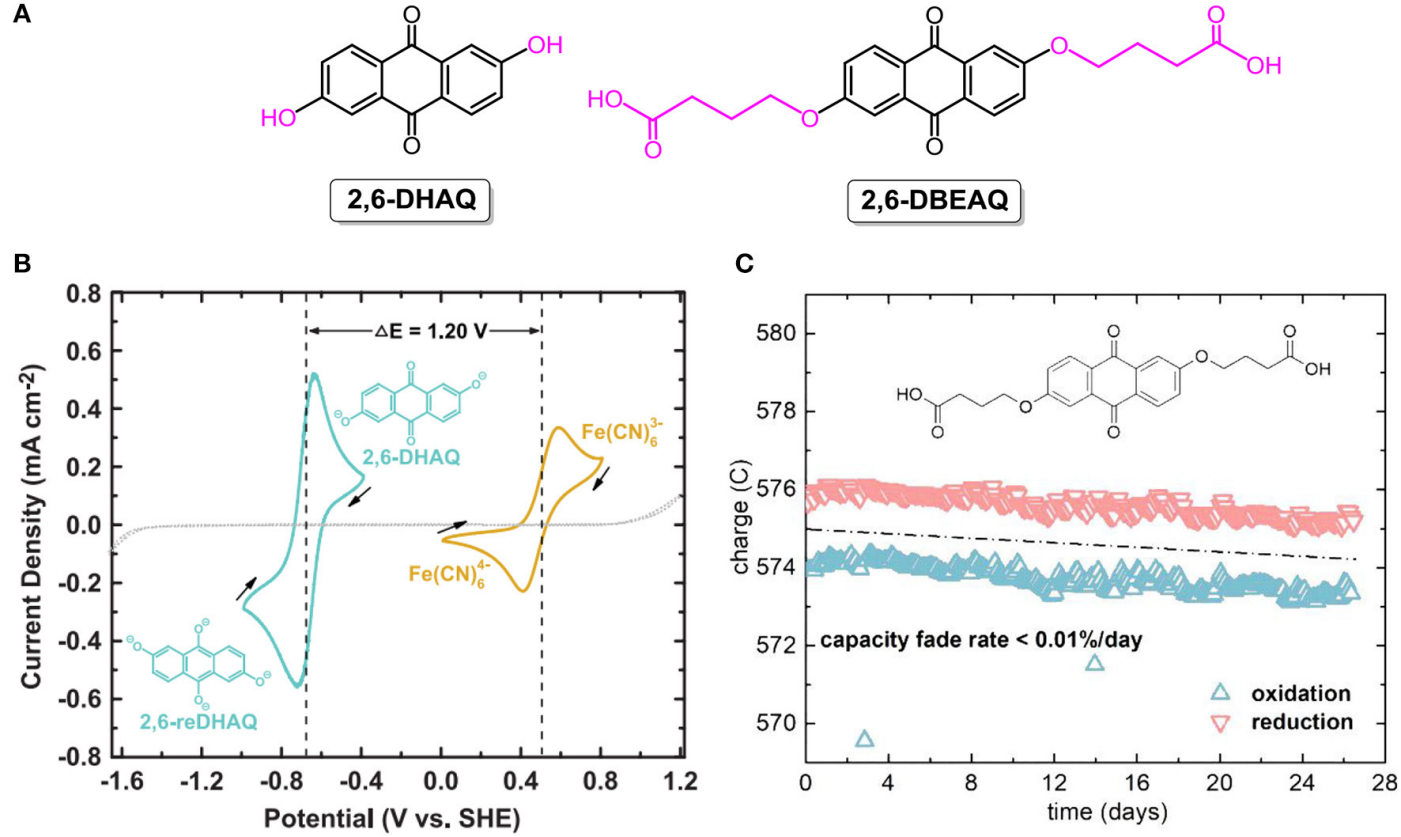
**(A)** Molecular structures of **2,6-DHAQ** and **2,6-DBEAQ**. **(B)** Cyclic voltammogram of 2 mM **2,6-DHAQ** (dark cyan curve) and ferrocyanide (gold curve) scanned at 100 mV/s on glassy carbon electrode; arrows indicate scan direction. Dotted line represents CV of 1 M KOH background scanned at 100 mV/s on graphite foil electrode. Reproduced with permission from ref. 76. Copyright 2015, American Association for the Advancement of Science. **(C)** Unbalanced compositionally symmetric cell cycling of 2,6-DBEAQ. Reproduced with permission from Kwabi et al. (2018). Copyright 2018, Elsevier.

To address concerns arising for acidic aqueous RFBs, organic electroactive molecules dissolved in alkaline supporting electrolyte were investigated as a promising alternative for storing massive energy. In 2015, the Aziz group demonstrated highly soluble quinone-based flow batteries in aqueous potassium hydroxide solutions. At the initial stage, commercially available 2,6-dihydroxyanthraquinone (**2,6-DHAQ**; the chemical structure is shown in Figure 7A) was used as one of the redox couples in alkaline aqueous RFBs. The open-circuit voltage of the **2,6-DHAQ**-based RFB reached 1.2 V (Figure 7B), and the capacity loss decreased to only 0.1% per cycle via the replacement of bromine with non-toxic ferricyanide as positive electrolyte (Lin et al., 2015). However, the energy output of the proposed RFB was limited by the poor solubility of highly stabilized **2,6-DHAQ**. To address this issue, a substituent with highly alkali-soluble carboxylate terminal groups was utilized to increase the solubility in alkaline electrolyte. The resulting compound, 4,4'-((9,10-anthraquinone-2,6-diyl)dioxy)dibutyrate (**2,6-DBEAQ**; the chemical structure is shown in Figure 7A), was six times more soluble than **2,6-DHAQ** at pH 12. At a pH value of as low as 12, an RFB assembled with **2,6-DBEAQ** as negative electrolyte exhibited a high open-circuit voltage of 1.05 V and a theoretical volumetric energy density of ca. 17 Wh L^−1^. Moreover, the symmetric cell with **2,6-DBEAQ** as both negative and positive electrolytes revealed a very low capacity fade rate (<0.01%/day and <0.001%/cycle, Figure 7C) (Kwabi et al., 2018).

Given above-stated advantages of anthraquinones, the use of benzoquinone and derivatives as analogs of anthraquinone is anticipated to be more promising in RFBs for large-scale energy storage, as they are inexpensive and a high theoretical energy density of 400 W h L^−1^ can be achieved from a redox reaction involving two electrons. However, it is reported that most benzoquinone-based acidic aqueous RFBs show low open-circuit voltage (<0.4 V) and poor cycling stability (Yang et al., 2014, 2016; Hoober-Burkhardt et al., 2017). To improve cell performance, benzoquinone derivatives were employed in alkaline aqueous RFBs, and advances were obtained. For example, an RFB working with 2,5-dihydroxy-1,4-benzoquinone as negative electrolyte showed an output voltage of 1.21 V, a peak galvanic power density of 300 mW cm^−2^, and a coulombic efficiency exceeding 99%, indicating that this would be a very promising direction for developing high-performance RFBs for practical applications (Yang et al., 2018b).

Besides the quinones, there are other organic electroactive molecules that have impressive performances in alkaline aqueous RFBs, such as alloxazine (Lin et al., 2016; Orita et al., 2016) and phenazine derivatives (Hollas et al., 2018). In 2016, Roy G. Gordon and co-workers reported a highly alkaline-soluble alloxazine-based organic electrolyte that enabled an aqueous RFB to exhibit an open-circuit voltage approaching 1.2 V with high current efficiency (99.7%) and capacity retention (99.98%) per cycle (Lin et al., 2016). The carboxylic group was introduced into the alloxazine derivative to increase the solubility in alkaline solutions, and the resulting alloxazine 7/8-carboxylic acid exhibited sufficiently high electrochemical and chemical stability, which opens up a new direction for designing organic electrolytes in aqueous RFBs. Later, Ying Shirley Meng and co-workers reported a biomimetic redox flow battery based on isoalloxazine, in which phosphate and hydroxy groups were used to enhance both the stabilization and solubility in alkaline solution. The isoalloxazine-based alkaline aqueous RFBs shows good cycling performance, with over 99% capacity retention over 100 cycles (Orita et al., 2016). With good redox properties and easily derivable structures, phenazine derivatives are promising organic anolytes for alkaline aqueous RFBs. In 2018, Wei Wang and co-workers reported a high-capacity alkaline aqueous RFB with phenazine-based organic electrolyte. Exceptionally high reversible anolyte capacity (67 Ah L^−1^) and capacity retention (99.98% per cycle over 500 cycles) have been achieved (Hollas et al., 2018). The pristine phenazine can hardly dissolve at all in aqueous solutions, but the solubility was increased to as much as 1.8 M by strategic modification with the hydroxy and sulfonic groups. Thus, tailoring functional groups is an effective strategy to improve both the solubility and stability of organic heterocyclic molecules, which usually show poor solubilities in aqueous solutions.

### Organic Electroactive Molecules for Neutral Aqueous RFBs

Improvements in the cell performance of aqueous RFBs haven been achieved through continuous efforts and have paved the way for practical application. However, RFBs systems employing strong acid and base have striking disadvantages, such as corrosivity, high maintenance cost, and environmental pollution hazard. Given the inherent merits such as being non-corrosive, non-flammable, environmentally benign and cheap, neutral aqueous supporting electrolytes (e.g., NaCl solution) in RFBs can effectively eliminate side reactions such as hydrogen evolution (common in acids), oxygen evolution (common in alkaline solutions), and other degradation of electroactive molecules catalyzed by acid and base (Beh et al., 2017; DeBruler et al., 2017; Ding and Yu, 2017; Hu et al., 2017).

**Figure 8:**
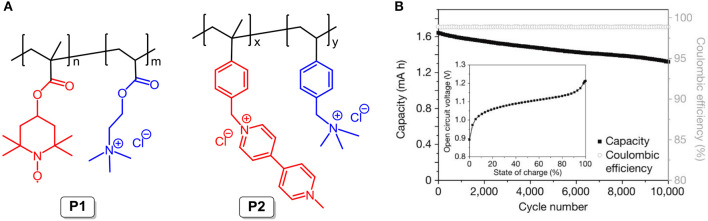
**(A)** Molecular structures of **P1** and **P2**. **(B)** The long-term stability of the polymer-based electrolytes was studied by repeated charge/discharge cycling over 10,000 cycles at 20 mA cm^−2^ in an unpumped test cell (inset) the open-circuit voltage of a polymer-based RFB as a function of the state of charge. Reproduced with permission from Janoschka et al. (2015). Copyright 2015, Springer Nature.

In 2015, the Schubert group reported an affordable, safe, and scalable neutral pH aqueous battery system with organic polymers as the electroactive materials. The TEMPO radical (**P1**; the chemical structure is shown in Figure 8A) and viologen derivative (**P2**; the chemical structure is shown in Figure 8A) were used as the cathode-active and anode-active molecules, respectively. Note that ammonium cation pendant groups were introduced to modify the backbones of both **P1** and **P2** to enhance their solubility in aqueous NaCl supporting solution. Compared to non-aqueous supporting electrolytes, the aqueous NaCl solution exhibits higher conductivity, lower cost, and great environmentally friendliness. Given the large molecular sizes of polymers, as stated above, the hydrodynamic radii of **P1** and **P2** are ~2 nm, larger than the pore sizes of the dialysis membrane (around 1 nm), and the permeability constants of the electroactive materials are remarkedly decreased. The cycling stability of the RFB supports the assumption that hardly any change occurred in the concentration of **P1** in the deficient compartment through migration through the membrane from the enriched side over 10,000 charging/discharging cycles, and this fits well with the undiminished coulombic efficiency (ca. 99%, Figure 8B) (Janoschka et al., 2015). Furthermore, the proposed RFB outputs an energy density of 10 W L^−1^ and maintains good performance at a current density reaching more than 100 mA cm^2^.

In combination with the flexible design of organic molecules, hydrophilic substituents like hydroxy groups were employed to modify the backbone of **TEMPO**. The resulting solubility of 4-hydroxy-tetramethylpiperidin-1-oxyl in water reaches a value as high as 2.1 M or more. By matching the TEMPO derivative with methyl viologen, a neutral aqueous RFB has been constructed and shows an exceptionally high cell voltage of 1.25 V, a high working current density of 100 mA cm^−2^, and nearly 100% coulombic efficiency within 100 cycles (Liu et al., 2016).

**Figure 9:**
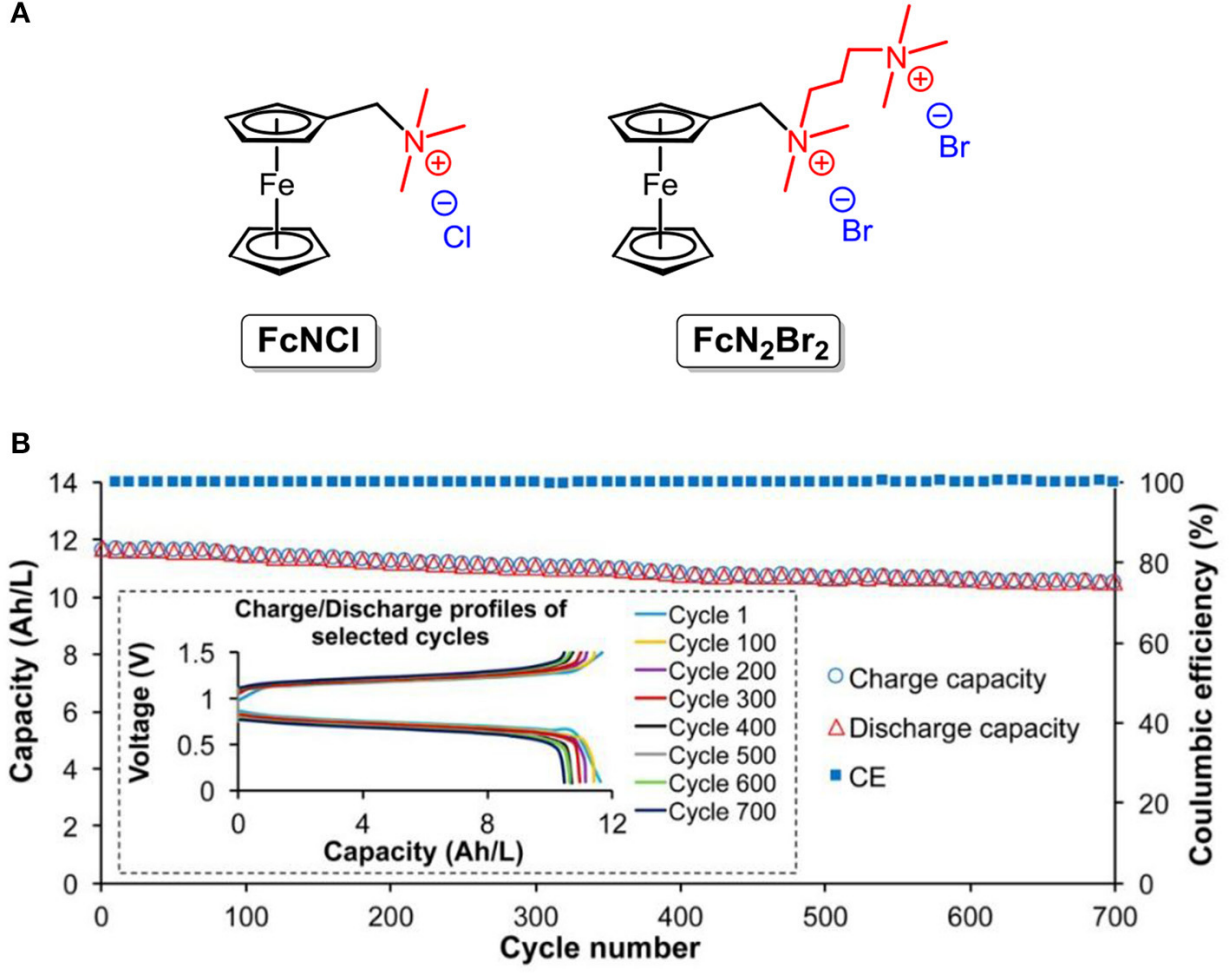
**(A)** Molecular structures of **FcNCl** and **FcN**_**2**_**Br**_**2**_. **(B)** Extended 700 cycle testing data of the 0.5 M **FcNCl**/**MV** aqueous RFB at 60 mA/cm^2^: capacity and coulombic efficiency vs. cycling numbers (for clarity, data points with an increment of 10 cycles were used for plotting) (inset) representative charge and discharge profiles of selected cycles. Reproduced with permission from Hu et al. (2017). Copyright 2017, American Chemical Society.

Note that ferrocene and derivatives have been adopted for energy storage in non-aqueous RFBs due to their poor solubility in water. Inspired by molecular synthesis approaches, the utilization of substituents with hydrophilic groups can effectively address the solubility concern. Liu and co-workers designed and synthesized two ferrocene derivatives (the chemical structures shown are in Figure 9A), namely ferrocenylmethyl trimethyl ammonium chloride (**FcNCl**) and *N*^1^-ferrocenylmethyl- *N*^1^, *N*^1^, *N*^2^, *N*^2^, *N*^2^-pentamethylpropane-1,2-diaminium dibromide (**FcN**_**2**_**Br**_**2**_). Under the hydrophilic effect of the tetraammonium moiety and halide counter ion, the solubilities of **FcNCl** and **FcN**_**2**_**Br**_**2**_ were increased to 4.0 and 3.1 M in water, respectively (Hu et al., 2017).

This strategy was also verified by reported work of the Aziz group, in which the solubility limit of bis((3-trimethylammonio)propyl)-ferrocene dichloride was enhanced to nearly 2 M (Beh et al., 2017). With high concentration and good redox reversibility, the **FcNCl**-based neutral aqueous RFB exhibits a power density of 125 mW cm^−2^ and 99.99% capacity retention per cycle (the structures are shown in Figure 9B) throughout a 700-cycle tests (Hu et al., 2017).

## Perspectives

The RFB has been recognized as the most promising electrochemical technology for large-scale energy storage, as such batteries can have the advantages of low cost, vast molecular diversity, highly tailorable properties, and high safety. However, some technical and economic challenges are still in urgent need of being issued before the widespread deployment of RFB systems at grid scale. Energy conversion between chemical energy and electric energy is achieved by redox reactions of electroactive materials at electrodes. The solubility limitation, electrochemical stability, permeability across a membrane, and cost of electroactive materials are crucial to the cell performance of an RFB and the capital cost. Compared to inorganic redox species (represented by metal ions), organic redox molecules, which can have inherent features such as flexible design, stable, easily tailored electrochemical properties, and cost-effectiveness, are more promising for RFBs targeted toward residential and industrial applications.

**Figure 10:**
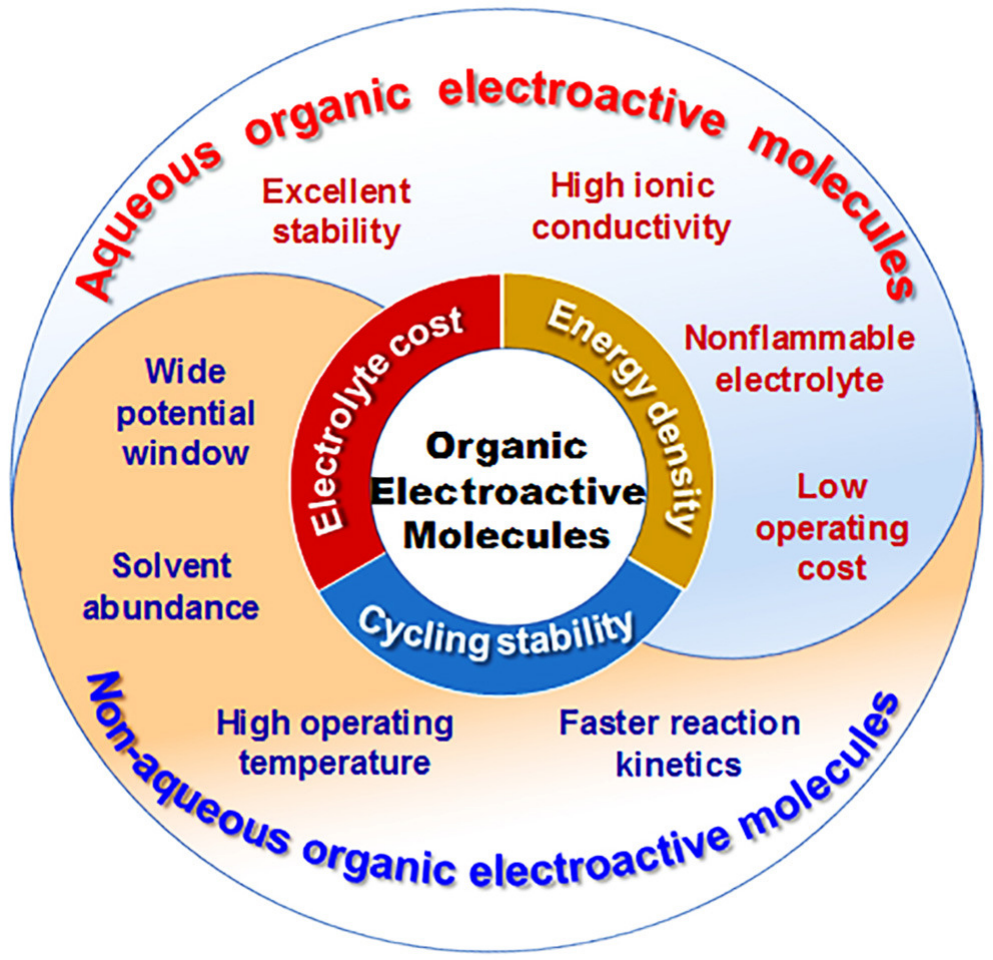
Correlation diagram of features of organic electroactive molecules in aqueous and non-aqueous RFBs, respectively.

RFBs with organic electroactive materials are categorized as aqueous and non-aqueous systems according to the supporting electrolytes. The features of organic electroactive molecules in aqueous and non-aqueous RFBs are summarized and shown in Figure 10. So far, aqueous RFBs are still dominating work targeted at practical applications due to their high ionic conductivity, excellent stability, low operating cost, and high safety as a result of not using hazardous or flammable solvent. However, an intrinsically low operational voltage window and poor energy density have become obstacles for the commercialization of aqueous RFBs. In contrast, non-aqueous RFBs exhibit wider redox potential windows and operating temperature ranges, as well as good flexibility through tuning both the physical and electrochemical properties of organic electroactive molecules. Moreover, the cell performance of non-aqueous RFBs can be dramatically improved with advances in molecule design. It is worth noting that low ionic conductivity, side reactions of organic electrolytes, and poor battery cycling performance have limited the wide-scale development of non-aqueous RFBs up until now.

It is well-known that the physicochemical and electrochemical properties of organic electroactive materials can be easily and effectively modified by functional groups. Herein, a series of prior results on tailoring organic redox molecules are summarized. Through improvements in solubility, stability, and safety, the proposed RFBs with those organic electroactive materials have achieved advances in cell performance. However, this field is still in its initial stage, as no superior RFB has been demonstrated to replace the well-developed all-vanadium RFB. Hopefully, this review will inspire more continuous interest and efforts in this field so as to encourage the development of more advanced RFBs with organic electroactive materials toward practical applications.

## Author Contributions

FZ wrote the main body of the manuscript. MY was responsible for the drawing of some figures and the formatting of the references. MD was in charge of the whole manuscript. CJ put forward the central idea of the manuscript and gives final modification. All authors contributed to the article and approved the submitted version.

## Conflict of Interest

The authors declare that the research was conducted in the absence of any commercial or financial relationships that could be construed as a potential conflict of interest.

## References

[B1] Armand M. Tarascon J. M. (2008). Building better batteries. Nature 451, 652–657. 10.1038/451652a 18256660

[B2] Bamgbopa M. O. Almheiri S. (2017). Influence of solvents on species crossover and capacity decay in non-aqueous vanadium redox flow batteries: Characterization of acetonitrile and 1, 3 dioxolane solvent mixture. J. Power Sources 342, 371–381. 10.1016/j.jpowsour.2016.12.050

[B3] Beh E. S. De Porcellinis D. Gracia R. L. Xia K. T. Gordon R. G. Aziz M. J. (2017). A Neutral pH aqueous organic–organometallic redox flow battery with extremely high capacity retention. ACS Energy Lett. 2, 639–644. 10.1021/acsenergylett.7b00019

[B4] Burgess M. Hernández-Burgos K. Schuh J. K. Davila J. Montoto E. C. Ewoldt R. H.. (2018). Modulation of the electrochemical reactivity of solubilized redox active polymers via polyelectrolyte dynamics. J. Am. Chem. Soc. 140, 2093–2104. 10.1021/jacs.7b08353 29369622

[B5] Burgess M. Moore J. S. Rodríguez-López J. (2016). Redox active polymers as soluble nanomaterials for energy storage. Acc. Chem. Res. 49, 2649–2657. 10.1021/acs.accounts.6b00341 27673336

[B6] Butler I. R. Thomas D. (2007). 6.05 - mononuclear iron compounds: ferrocenes, in Comprehensive Organometallic Chemistry III, eds Mingos D. M. P. Crabtree R. H. (Oxford: Elsevier), 185–220.

[B7] Cappillino P. J. Pratt Iii H. D. Hudak N. S. Tomson N. C. Anderson T. M. Anstey M. R. (2014). Application of redox non-innocent ligands to non-aqueous flow battery electrolytes. Adv. Energy Mater. 4:1300566 10.1002/aenm.201300566

[B8] Carney T. J. Collins S. J. Moore J. S. Brushett F. R. (2017). Concentration-dependent dimerization of anthraquinone disulfonic acid and its impact on charge storage. Chem. Mater. 29, 4801–4810. 10.1021/acs.chemmater.7b00616

[B9] Chakrabarti M. H. Dryfe R. A. W. Roberts E. P. L. (2007). Evaluation of electrolytes for redox flow battery applications. Electrochim. Acta 52, 2189–2195. 10.1016/j.electacta.2006.08.052

[B10] Chang S. Ye J. Zhou W. Wu C. Ding M. Long Y. (2019). A low-cost SPEEK-K type membrane for neutral aqueous zinc-iron redox flow battery. Surf. Coat. Technol. 358, 190–194. 10.1016/j.surfcoat.2018.11.028

[B11] Chen J. Li L. Wu L. Yao Q. Yang H. Liu Z. (2018). Enhanced cycle stability of Na_0.9_Ni_0.45_Mn_0.55_O_2_ through tailoring O3/P2 hybrid structures for sodium-ion batteries. J. Power Sources 406, 110–117. 10.1016/j.jpowsour.2018.10.058

[B12] Chen J. Yang H. Li T. Liu C. Tong H. Chen J.. (2019). The effects of reversibility of H_2_-H_3_ phase transition on Ni-rich layered oxide cathode for high-energy lithium-ion batteries. Front. Chem. 7:500. 10.3389/fchem.2019.00500 31380345 PMC6646592

[B13] Chen Z. Xu M. Zhu H. Xie T. Wang W. Zhao Q. (2013). Enhanced electrochemical performance of polyacene coated LiMn_2_O_3.95_F_0.05_ for lithium ion batteries. Appl. Surf. Sci. 286, 177–183. 10.1016/j.apsusc.2013.09.044

[B14] Chen Z. Yan X. Xu M. Cao K. Zhu H. Li L.. (2017). Building honeycomb-like hollow microsphere architecture in a bubble template reaction for high-performance lithium-rich layered oxide cathode materials. ACS Appl. Mater. Interf. 9, 30617–30625. 10.1021/acsami.7b07542 28828854

[B15] Cosimbescu L. Wei X. Vijayakumar M. Xu W. Helm M. L. Burton S. D.. (2015). Anion-tunable properties and electrochemical performance of functionalized ferrocene compounds. Sci. Rep. 5:14117. 10.1038/srep14117 26374254 PMC4571638

[B16] Dai L.-X. Hou X.-L. (2010). Chiral Ferrocenes in Asymmetric Catalysis: Synthesis and Applications. Weinheim: John Wiley and Sons Ltd (2010).

[B17] Darling R. M. Gallagher K. G. Kowalski J. A. Ha S. Brushett F. R. (2014). Pathways to low-cost electrochemical energy storage: a comparison of aqueous and nonaqueous flow batteries. Energy Environ. Sci. 7, 3459–3477. 10.1039/C4EE02158D

[B18] DeBruler C. Hu B. Moss J. Liu X. Luo J. Sun Y. (2017). Designer two-electron storage viologen anolyte materials for neutral aqueous organic redox flow batteries. Chemistry 3, 961–978. 10.1016/j.chempr.2017.11.001

[B19] Ding C. Zhang H. Li X. Liu T. Xing F. (2013). Vanadium flow battery for energy storage: prospects and challenges. J. Phys. Chem. Lett. 4, 1281–1294. 10.1021/jz4001032 26282141

[B20] Ding M. Hickey A. K. Pink M. Telser J. Tierney D. L. Amoza M.. (2019). Magnetization slow dynamics in ferrocenium complexes. Chem. A Eur. J. 25, 10625–10632. 10.1002/chem.201900799 31066934

[B21] Ding Y. Li Y. Yu G. (2016). Exploring bio-inspired quinone-based organic redox flow batteries: a combined experimental and computational study. Chemistry 1, 790–801. 10.1016/j.chempr.2016.09.004

[B22] Ding Y. Yu G. (2017). The promise of environmentally benign redox flow batteries by molecular engineering. Angewan. Chem. Int. Edn. 56, 8614–8616. 10.1002/anie.201701254 28387026

[B23] Ding Y. Zhang C. Zhang L. Zhou Y. Yu G. (2018). Molecular engineering of organic electroactive materials for redox flow batteries. Chem. Soc. Rev. 47, 69–103. 10.1039/C7CS00569E 29044260

[B24] Dunn B. Kamath H. Tarascon J.-M. (2011). Electrical energy storage for the grid: a battery of choices. Science 334, 928–935. 10.1126/science.1212741 22096188

[B25] Er S. Suh C. Marshak M. P. Aspuru-Guzik A. (2015). Computational design of molecules for an all-quinone redox flow battery. Chem. Sci. 6, 885–893. 10.1039/C4SC03030C 29560173 PMC5811157

[B26] Gerhardt M. R. Tong L. Gómez-Bombarelli R. Chen Q. Marshak M. P. Galvin C. J. (2017). Anthraquinone derivatives in aqueous flow batteries. Adv. Energy Mater. 7:1601488 10.1002/aenm.201601488

[B27] Goulet M.-A. Aziz M. J. (2018). Flow battery molecular reactant stability determined by symmetric cell cycling methods. J. Electrochem. Soc. 165:A1466–A1477. 10.1149/2.0891807jes

[B28] Häupler B. Wild A. Schubert U. S. (2015). Carbonyls: powerful organic materials for secondary batteries. Adv. Energy Mater. 5:1402034 10.1002/aenm.201402034

[B29] Herr T. Noack J. Fischer P. Tübke J. (2013). 1,3-Dioxolane, tetrahydrofuran, acetylacetone and dimethyl sulfoxide as solvents for non-aqueous vanadium acetylacetonate redox-flow-batteries. Electrochim. Acta 113, 127–133. 10.1016/j.electacta.2013.09.055

[B30] Holdren J. P. (2007). Energy and sustainability. Science 315, 737–737. 10.1126/science.1139792 17289943

[B31] Hollas A. Wei X. Murugesan V. Nie Z. Li B. Reed D. (2018). A biomimetic high-capacity phenazine-based anolyte for aqueous organic redox flow batteries. Nature Energy 3, 508–514. 10.1038/s41560-018-0167-3

[B32] Hoober-Burkhardt L. Krishnamoorthy S. Yang B. Murali A. Nirmalchandar A. Prakash G. K. S. (2017). A new michael-reaction-resistant benzoquinone for aqueous organic redox flow batteries. J. Electrochem. Soc. 164, A600–A607. 10.1149/2.0351704jes

[B33] Hu B. DeBruler C. Rhodes Z. Liu T. L. (2017). Long-cycling aqueous organic redox flow battery (AORFB) toward sustainable and safe energy storage. J. Am. Chem. Soc. 139, 1207–1214. 10.1021/jacs.6b10984 27973765

[B34] Huskinson B. Marshak M. P. Suh C. Er S. Gerhardt M. R. Galvin C. J.. (2014). A metal-free organic–inorganic aqueous flow battery. Nature 505, 195–198. 10.1038/nature12909 24402280

[B35] Iyer V. A. Schuh J. K. Montoto E. C. Pavan Nemani V. Qian S. Nagarjuna G. (2017). Assessing the impact of electrolyte conductivity and viscosity on the reactor cost and pressure drop of redox-active polymer flow batteries. J. Power Sources 361, 334–344. 10.1016/j.jpowsour.2017.06.052

[B36] Janoschka T. Martin N. Martin U. Friebe C. Morgenstern S. Hiller H.. (2015). An aqueous, polymer-based redox-flow battery using non-corrosive, safe, and low-cost materials. Nature 527, 78–81. 10.1038/nature15746 26503039

[B37] Jing Y. Liang Y. Gheytani S. Yao Y. (2017). Cross-conjugated oligomeric quinones for high performance organic batteries. Nano Energy 37, 46–52. 10.1016/j.nanoen.2017.04.055

[B38] Kamat P. V. Schanze K. S. Buriak J. M. (2017). Redox flow batteries. ACS Energy Lett. 2, 1368–1369. 10.1021/acsenergylett.7b00361

[B39] Kealy T. J. Pauson P. L. (1951). A new type of organo-iron compound. Nature 168, 1039–1040. 10.1038/1681039b0

[B40] Kim H.-s. Yoon T. Kim Y. Hwang S. Ryu J. H. Oh S. M. (2016). Increase of both solubility and working voltage by acetyl substitution on ferrocene for non-aqueous flow battery. Electrochem. Commun. 69, 72–75. 10.1016/j.elecom.2016.06.002

[B41] Kim J.-H. Kim K. J. Park M.-S. Lee N. J. Hwang U. Kim H. (2011). Development of metal-based electrodes for non-aqueous redox flow batteries. Electrochem. Commun. 13, 997–1000. 10.1016/j.elecom.2011.06.022

[B42] Kwabi D. G. Ji Y. Aziz M. J. (2020). Electrolyte lifetime in aqueous organic redox flow batteries: a critical review. Chem. Rev. [Epub ahead of print]. 10.1021/acs.chemrev.9b00599 32053366

[B43] Kwabi D. G. Lin K. Ji Y. Kerr E. F. Goulet M.-A. De Porcellinis D. (2018). Alkaline quinone flow battery with long lifetime at pH 12. Joule 2, 1894–1906. 10.1016/j.joule.2018.07.005

[B44] Lai Y. Y. Li X. Zhu Y. (2020). Polymeric active materials for redox flow battery application. ACS Appl. Polymer Mater. 2, 113–128. 10.1021/acsapm.9b00864

[B45] Leadbetter J. Swan L. G. (2012). Selection of battery technology to support grid-integrated renewable electricity. J. Power Sources 216, 376–386. 10.1016/j.jpowsour.2012.05.081

[B46] Leung P. Shah A. A. Sanz L. Flox C. Morante J. R. Xu Q. (2017). Recent developments in organic redox flow batteries: a critical review. J. Power Sources 360, 243–283. 10.1016/j.jpowsour.2017.05.057

[B47] Li G. Jia Y. Zhang S. Li X. Li J. Li L. (2017). The crossover behavior of bromine species in the metal-free flow battery. J. Appl. Electrochem. 47, 261–272. 10.1007/s10800-016-1033-2

[B48] Li L. Chen Z. Song L. Xu M. Zhu H. Gong L. (2015a). Characterization and electrochemical performance of lithium-active titanium dioxide inlaid LiNi_0.5_Co_0.2_Mn_0.3_O_2_ material prepared by lithium residue-assisted method. J. Alloys Compd. 638, 77–82. 10.1016/j.jallcom.2015.03.071

[B49] Li L. Chen Z. Zhang Q. Xu M. Zhou X. Zhu H. (2015b). A hydrolysis-hydrothermal route for the synthesis of ultrathin LiAlO_2_-inlaid LiNi_0.5_Co_0.2_Mn_0.3_O_2_ as a high-performance cathode material for lithium ion batteries. J. Mater. Chem. A 3, 894–904. 10.1039/C4TA05902F

[B50] Li L. Xu M. Chen Z. Zhou X. Zhang Q. Zhu H. (2015c). High-performance lithium-rich layered oxide materials: Effects of chelating agents on microstructure and electrochemical properties. Electrochim. Acta 174, 446–455. 10.1016/j.electacta.2015.05.171

[B51] Li L. Xu M. Yao Q. Chen Z. Song L. Zhang Z.. (2016a). Alleviating surface degradation of nickel-rich layered oxide cathode material by encapsulating with nanoscale Li-ions/electrons superionic conductors hybrid membrane for advanced Li-ion batteries. ACS Appl. Mater. Interfaces 8, 30879–30889. 10.1021/acsami.6b09197 27805812

[B52] Li L. Yao Q. Chen Z. Song L. Xie T. Zhu H. (2015d). Effects of lithium-active manganese trioxide coating on the structural and electrochemical characteristics of LiNi_0.5_Co_0.2_Mn_0.3_O_2_ as cathode materials for lithium ion battery. J. Alloys Compd. 650, 684–691. 10.1016/j.jallcom.2015.08.041

[B53] Li L. Yao Q. Zhu H. Chen Z. Song L. Duan J. (2016b). Effect of Al substitution sites on Li_1−x_Al_x_(Ni_0.5_Co_0.2_Mn_0.3_)_1−y_Al_y_O_2_ cathode materials for lithium ion batteries. J Alloys Compd. 686, 30–37. 10.1016/j.jallcom.2016.05.333

[B54] Li Z. Li S. Liu S. Huang K. Fang D. Wang F. (2011). Electrochemical properties of an all-organic redox flow battery using 2,2,6,6-tetramethyl-1-piperidinyloxy and N-methylphthalimide. Electrochem. Solid State Lett. 14, A171–A173. 10.1149/2.012112esl

[B55] Lin K. Chen Q. Gerhardt M. R. Tong L. Kim S. B. Eisenach L.. (2015). Alkaline quinone flow battery. Science 349, 1529–1532. 10.1126/science.aab3033 26404834

[B56] Lin K. Gómez-Bombarelli R. Beh E. S. Tong L. Chen Q. Valle A. (2016). A redox-flow battery with an alloxazine-based organic electrolyte. Nat. Energy 1, 1–8. 10.1038/nenergy.2016.102

[B57] Liu K. Yang J. Liang S. Hou H. Chen Y. Wang J.. (2018). An emission-free vacuum chlorinating process for simultaneous sulfur fixation and lead recovery from spent lead-acid batteries. Environ. Sci. Technol. 52, 2235–2241. 10.1021/acs.est.7b05283 29338210

[B58] Liu Q. Shinkle A. A. Li Y. Monroe C. W. Thompson L. T. Sleightholme A. E. S. (2010). Non-aqueous chromium acetylacetonate electrolyte for redox flow batteries. Electrochem. Commun. 12, 1634–1637. 10.1016/j.elecom.2010.09.013

[B59] Liu T. Wei X. Nie Z. Sprenkle V. Wang W. (2016). A total organic aqueous redox flow battery employing a low cost and sustainable methyl viologen anolyte and 4-HO-TEMPO catholyte. Adv. Energy Mater. 6:1501449 10.1002/aenm.201501449

[B60] Lou X. Ye J. Xia L. Chang S. Zhao X. Wu C.. (2019). Highly efficient and low cost SPEEK/TiO_2_ nanocomposite membrane for vanadium redox flow battery. J. Nanosci. Nanotechnol. 19, 2247–2252. 10.1166/jnn.2019.16467 30486977

[B61] Luo J. Hu B. Hu M. Zhao Y. Liu T. L. (2019). Status and prospects of organic redox flow batteries toward sustainable energy storage. ACS Energy Lett. 4, 2220–2240. 10.1021/acsenergylett.9b01332

[B62] Ma C. Shu Y. Chen H. (2015). Recycling lead from spent lead pastes using oxalate and sodium oxalate and preparation of novel lead oxide for lead-acid batteries. RSC Adv. 5, 94895–94902. 10.1039/C5RA18627G

[B63] Matsuda Y. Tanaka K. Okada M. Takasu Y. Morita M. Matsumura-Inoue T. (1988). A rechargeable redox battery utilizing ruthenium complexes with non-aqueous organic electrolyte. J. Appl. Electrochem. 18, 909–914. 10.1007/BF01016050

[B64] Milshtein J. D. Barton J. L. Darling R. M. Brushett F. R. (2016). 4-acetamido-2,2,6,6-tetramethylpiperidine-1-oxyl as a model organic redox active compound for nonaqueous flow batteries. J. Power Sources 327, 151–159. 10.1016/j.jpowsour.2016.06.125

[B65] Montoto E. C. Cao Y. Hernández-Burgos K. Sevov C. S. Braten M. N. Helms B. A. (2018). Effect of the backbone tether on the electrochemical properties of soluble cyclopropenium redox-active polymers. Macromolecules 51, 3539–3546. 10.1021/acs.macromol.8b00574

[B66] Montoto E. C. Nagarjuna G. Moore J. S. Rodríguez-López J. (2017). Redox active polymers for non-aqueous redox flow batteries: validation of the size-exclusion approach. J. Electrochem. Soc. 164, A1688–A1694. 10.1149/2.1511707jes

[B67] Nagarjuna G. Hui J. Cheng K. J. Lichtenstein T. Shen M. Moore J. S.. (2014). Impact of redox-active polymer molecular weight on the electrochemical properties and transport across porous separators in nonaqueous solvents. J. Am. Chem. Soc. 136, 16309–16316. 10.1021/ja508482e 25325703

[B68] Nishide H. Iwasa S. Pu Y.-J. Suga T. Nakahara K. Satoh M. (2004). Organic radical battery: nitroxide polymers as a cathode-active material. Electrochim. Acta 50, 827–831. 10.1016/j.electacta.2004.02.05217457423

[B69] Orita A. Verde M. G. Sakai M. Meng Y. S. (2016). A biomimetic redox flow battery based on flavin mononucleotide. Nat. Commun. 7, 1–8. 10.1038/ncomms13230 27767026 PMC5078740

[B70] Park M. Ryu J. Wang W. Cho J. (2017). Material design and engineering of next-generation flow-battery technologies. Nat. Rev. Mater. 2:16080 10.1038/natrevmats.2016.80

[B71] Park M. Shin D.-S. Ryu J. Choi M. Park N. Hong S. Y.. (2015). Organic-catholyte-containing flexible rechargeable lithium batteries. Adv. Mater. 27, 5141–5146. 10.1002/adma.201502329 26237211

[B72] Phillips E. S. (2011). Ferrocenes: Compounds, Properties and Applications. New York, NY: Nova Science Publishers.

[B73] Qin W. Li J. Liu X. Zhou N. Wu C. Ding M.. (2019). Formation of needle-like porous CoNi2S4-MnOOH for high performance hybrid supercapacitors with high energy density. J. Coll. Interface Sci. 554, 125–132. 10.1016/j.jcis.2019.07.010 31288176

[B74] Quan M. Sanchez D. Wasylkiw M. F. Smith D. K. (2007). Voltammetry of quinones in unbuffered aqueous solution: reassessing the roles of proton transfer and hydrogen bonding in the aqueous electrochemistry of quinones. J. Am. Chem. Soc. 129, 12847–12856. 10.1021/ja0743083 17910453

[B75] Rajagopalan R. Tang Y. Ji X. Jia C. Wang H. (2020a). Advancements and challenges in potassium ion batteries: a comprehensive review. Adv. Funct. Mater. 30:1909486 10.1002/adfm.201909486

[B76] Rajagopalan R. Tang Y. Jia C. Ji X. Wang H. (2020b). Understanding the sodium storage mechanisms of organic electrodes in sodium ion batteries: issues and solutions. Energy Environ. Sci. [Epub ahead of print]. 10.1039/C9EE03637G

[B77] Schon T. B. McAllister B. T. Li P.-F. Seferos D. S. (2016). The rise of organic electrode materials for energy storage. Chem. Soc. Rev. 45, 6345–6404. 10.1039/C6CS00173D 27273252

[B78] Scott D. T. McKnight D. M. Blunt-Harris E. L. Kolesar S. E. Lovley D. R. (1998). Quinone moieties act as electron acceptors in the reduction of humic substances by humics-reducing microorganisms. Environ. Sci. Technol. 32, 2984–2989. 10.1021/es980272q

[B79] Sevov C. S. Fisher S. L. Thompson L. T. Sanford M. S. (2016). Mechanism-based development of a low-potential, soluble, and cyclable multielectron anolyte for nonaqueous redox flow batteries. J. Am. Chem. Soc. 138, 15378–15384. 10.1021/jacs.6b07638 27933936

[B80] Sleightholme A. E. S. Shinkle A. A. Liu Q. Li Y. Monroe C. W. Thompson L. T. (2011). Non-aqueous manganese acetylacetonate electrolyte for redox flow batteries. J. Power Sources 196, 5742–5745. 10.1016/j.jpowsour.2011.02.020

[B81] Soloveichik G. L. (2015). Flow batteries: current status and trends. Chem. Rev. 115, 11533–11558. 10.1021/cr500720t 26389560

[B82] Štěpnička S. (2008). Ferrocenes: Ligands, Materials and Biomolecules, Chichester: John Wiley & Sons, Ltd.

[B83] Takechi K. Kato Y. Hase Y. (2015). A highly concentrated catholyte based on a solvate ionic liquid for rechargeable flow batteries. Adv. Mater. 27, 2501–2506. 10.1002/adma.201405840 25757722

[B84] Turner J. A. (1999). A realizable renewable energy future. Science 285, 687–689. 10.1126/science.285.5428.687 10426982

[B85] Wei D. Zhong S. Hang Z. Zhang X. Zhu C. Duan J. (2018). In situ construction of interconnected SnO_2_/nitrogen-doped Carbon@TiO_2_ networks for lithium-ion half/full cells. Electrochim. Acta 290, 312–321. 10.1016/j.electacta.2018.08.094

[B86] Wei X. Cosimbescu L. Xu W. Hu J. Z. Vijayakumar M. Feng J. (2015). Towards high-performance nonaqueous redox flow electrolyte via ionic modification of active species. Adv. Energy Mater. 5:1400678 10.1002/aenm.201400678

[B87] Wei X. Xu W. Vijayakumar M. Cosimbescu L. Liu T. Sprenkle V.. (2014). TEMPO-based catholyte for high-energy density nonaqueous redox flow batteries. Adv. Mater. 26, 7649–7653. 10.1002/adma.201403746 25327755

[B88] Wu C. Chen L. Lou X. Ding M. Jia C. (2018a). Fabrication of cobalt-nickel-zinc ternary oxide nanosheet and applications for supercapacitor electrode. Front. Chem. 6:597. 10.3389/fchem.2018.00597 30555822 PMC6281991

[B89] Wu C. Lou X. Jia C. (2019a). Porous Ni-Mo-Co hydroxide nanoflakes on carbon cloth for supercapacitor application. J. Nanosci. Nanotechnol. 19, 272–276. 10.1166/jnn.2019.16450 30327036

[B90] Wu C. Zhu Y. Ding M. Jia C. Zhang K. (2018b). Fabrication of plate-like MnO2 with excellent cycle stability for supercapacitor electrodes. Electrochim. Acta 291, 249–255. 10.1016/j.electacta.2018.08.126

[B91] Wu C. Zhu Y. Guan C. Jia C. Qin W. Wang X. (2019b). Mesoporous aluminium manganese cobalt oxide with pentahedron structures for energy storage devices. J. Mater. Chem. A 7, 18417–18427. 10.1039/C9TA06319F

[B92] Xia L. Zhang Q. Wu C. Liu Y. Ding M. Ye J. (2019). Graphene coated carbon felt as a high-performance electrode for all vanadium redox flow batteries. Surf. Coat. Technol. 358, 153–158. 10.1016/j.surfcoat.2018.11.024

[B93] Xing X. Zhang D. Li Y. (2015). A non-aqueous all-cobalt redox low battery using 1,10-phenanthrolinecobalt(II) hexafluorophosphate as active species. J. Power Sources 279, 205–209. 10.1016/j.jpowsour.2015.01.011

[B94] Xu M. Chen Z. Zhu H. Yan X. Li L. Zhao Q. (2015). Mitigating capacity fade by constructing highly ordered mesoporous Al_2_O_3_/polyacene double-shelled architecture in Li-rich cathode materials. J. Mater. Chem. A 3, 13933–13945. 10.1039/C5TA03676C

[B95] Xu Y. Wen Y.-H. Cheng J. Cao G.-P. Yang Y.-S. (2010). A study of tiron in aqueous solutions for redox flow battery application. Electrochim. Acta 55, 715–720. 10.1016/j.electacta.2009.09.031

[B96] Yang B. Hoober-Burkhardt L. Krishnamoorthy S. Murali A. Prakash G. K. S. Narayanan S. R. (2016). High-performance aqueous organic flow battery with quinone-based redox couples at both electrodes. J. Electrochem. Soc. 163, A1442–A1449. 10.1149/2.1371607jes

[B97] Yang B. Hoober-Burkhardt L. Wang F. Surya Prakash G. K. Narayanan S. R. (2014). An inexpensive aqueous flow battery for large-scale electrical energy storage based on water-soluble organic redox couples. J. Electrochem. Soc. 161, A1371–A1380. 10.1149/2.1001409jes

[B98] Yang H. Wu H.-H. Ge M. Li L. Yuan Y. Yao Q.. (2019). Simultaneously dual modification of Ni-rich layered oxide cathode for high-energy lithium-ion batteries. Adv. Funct. Mater. 29:1808825. 10.1002/adfm.20180882531380345

[B99] Yang S. Li R. Cai X. Xue K. Yang B. Hu X. (2018a). Enhanced cycle performance and lifetime estimation of lead-acid batteries. N. J. Chem. 42, 8900–8904. 10.1039/C8NJ00542G

[B100] Yang Z. Tong L. Tabor D. P. Beh E. S. Goulet M.-A. De Porcellinis D. (2018b). Alkaline benzoquinone aqueous flow battery for large-scale storage of electrical energy. Adv. Energy Mater. 8:1702056 10.1002/aenm.201702056

[B101] Yang Z. Zhang J. Kintner-Meyer M. C. W. Lu X. Choi D. Lemmon J. P.. (2011). Electrochemical energy storage for green grid. Chem. Rev. 111, 3577–3613. 10.1021/cr100290v 21375330

[B102] Ye J. Cheng Y. Sun L. Ding M. Wu C. Yuan D. (2019a). A green SPEEK/lignin composite membrane with high ion selectivity for vanadium redox flow battery. J. Membr. Sci. 572, 110–118. 10.1016/j.memsci.2018.11.009

[B103] Ye J. Lou X. Wu C. Wu S. Ding M. Sun L.. (2018). Ion selectivity and stability enhancement of SPEEK/lignin membrane for vanadium redox flow battery: the degree of sulfonation effect. Front. Chem. 6:549. 10.3389/fchem.2018.00549 30483496 PMC6240590

[B104] Ye J. Wu C. Qin W. Zhong F. Ding M. (2020). Advanced sulfonated poly(ether ether ketone)/graphene-oxide/titanium dioxide nanoparticle composited membrane with superior cyclability for vanadium redox flow battery. J. Nanosci. Nanotechnol. 20, 4714–4721. 10.1166/jnn.2020.18503 32126646

[B105] Ye J. Xia L. Wu C. Ding M. Jia C. Wang Q. (2019b). Redox targeting-based flow batteries. J. Phys. D Appl. Phys. 52:443001. 10.1088/1361-6463/ab325130118550

[B106] Zeng L. Lou X. Zhang J. Wu C. Liu J. Jia C. (2019). Carbonaceous mudstone and lignin-derived activated carbon and its application for supercapacitor electrode. Surf. Coat. Technol. 357, 580–586. 10.1016/j.surfcoat.2018.10.041

[B107] Zhang H. Lu W. Li X. (2019). Progress and perspectives of flow battery technologies. Electrochem. Energy Rev. 2, 492–506. 10.1007/s41918-019-00047-1

